# Severe Gastrointestinal Intolerance After Resuming Maintenance-Dose Semaglutide Following Ramadan-Related Treatment Interruption: A Case Report

**DOI:** 10.7759/cureus.113026

**Published:** 2026-07-20

**Authors:** Hafiza Valizada, Mihail Zilbermint

**Affiliations:** 1 Department of Endocrinology, Diabetes and Metabolism, Ellab Diagnostic, Baku, AZE; 2 Department of Hospital Based Medicine, Johns Hopkins Community Physicians, Johns Hopkins Medicine, Baltimore, USA; 3 Department of Endocrinology, Diabetes, and Metabolism, Johns Hopkins University School of Medicine, Baltimore, USA; 4 Department of Medicine, Suburban Hospital, Johns Hopkins Medicine, Bethesda, USA

**Keywords:** drug reinitiation, gastrointestinal intolerance, glp-1 receptor agonists, glucagon-like peptide-1 receptor agonists, ramadan, semaglutide, treatment interruption

## Abstract

Glucagon-like peptide-1 (GLP-1) receptor agonists are widely used in the treatment of obesity and diabetes mellitus because of their efficacy in weight reduction, glycemic control, and overall metabolic benefits. Gastrointestinal adverse events are among the most frequently reported side effects associated with these agents and are typically transient, occurring predominantly during dose escalation. However, severe gastrointestinal intolerance requiring hospitalization has rarely been reported.

We present the case of a 51-year-old woman with obesity, hypertension, hyperlipidemia, and anxiety who developed severe nausea, vomiting, and diarrhea one day after restarting semaglutide 2.4 mg following a five-week treatment interruption during Ramadan. The patient had previously tolerated semaglutide therapy for approximately one year, with successful weight loss. Laboratory evaluation revealed significant electrolyte abnormalities, while contrast-enhanced abdominal and pelvic CT showed no acute pathology. The patient was managed with supportive treatment, including intravenous hydration, electrolyte replacement, antiemetics, and dietary modifications. She was discharged after four days of hospitalization, and cautious semaglutide reinitiation with dose escalation was planned after two weeks.

This case highlights an important educational opportunity during Ramadan counseling, as patients receiving GLP-1 receptor agonists should generally be advised not to discontinue therapy solely because of daytime fasting unless clinically indicated. If treatment interruption occurs for a prolonged period, cautious reinitiation beginning at a lower dose with gradual dose escalation should be considered to minimize gastrointestinal adverse events. Careful dose re-titration after extended lapses in therapy may help reduce this risk. Further studies are needed to establish evidence-based recommendations for semaglutide reinitiation after prolonged treatment interruptions.

## Introduction

Glucagon-like peptide-1 (GLP-1) receptor agonists are increasingly used in the management of obesity and type 2 diabetes mellitus because of their efficacy in improving glycemic control and promoting weight loss, as well as their demonstrated cardiovascular and renal benefits [1]. Semaglutide shares approximately 94% structural homology with native human GLP-1 but contains specific modifications designed to prolong its half-life, enabling once-weekly administration [2]. These agents improve glycemic control by enhancing pancreatic β-cell function and suppressing glucagon secretion from pancreatic α-cells, thereby reducing hepatic glucose production. In addition, they slow gastric emptying and reduce appetite through central mechanisms, contributing to weight loss and improved metabolic control [3].

Gastrointestinal adverse events, including nausea, vomiting, constipation, diarrhea, and abdominal discomfort, are among the commonly reported side effects of GLP-1 receptor agonist therapy and may adversely affect treatment adherence and tolerability [4]. Delayed gastric emptying is considered a major mechanism underlying these gastrointestinal adverse effects [1,5]. The GLP-1 receptor agonists are also thought to induce nausea and vomiting through activation of central and peripheral GLP-1 receptor pathways involved in gut-brain signaling and regulation of emesis [5]. To improve gastrointestinal tolerability, semaglutide is initiated at a low dose and gradually escalated to the maintenance dose according to the recommended dosing schedule [6].

During Ramadan, some patients may temporarily interrupt GLP-1 receptor agonist therapy while fasting, despite these agents generally being considered safe during Ramadan [7,8]. Evidence to guide semaglutide reinitiation after prolonged treatment interruption remains limited [9]. This lack of evidence creates uncertainty regarding the optimal strategy for treatment reinitiation, particularly whether semaglutide should be resumed at the previous maintenance dose or reinitiated using gradual dose escalation [9]. Although mild gastrointestinal adverse effects are commonly observed during the dose-escalation phase, severe gastrointestinal intolerance requiring hospitalization has rarely been reported. Herein, we present a case of severe gastrointestinal intolerance requiring hospitalization following reinitiation of semaglutide at the maintenance dose after a Ramadan-related treatment interruption.

## Case presentation

A 51-year-old woman presented to the emergency department with severe nausea, vomiting, and diarrhea that developed one day after subcutaneous administration of semaglutide 2.4 mg. Her past medical history was significant for obesity, hypertension, hyperlipidemia, and anxiety. The patient had been using semaglutide for approximately one year and had achieved a weight loss of 15 pounds during treatment. She had previously tolerated the maintenance dose of 2.4 mg without significant gastrointestinal adverse effects. However, she had independently discontinued semaglutide for approximately five weeks during Ramadan because of concerns regarding reduced oral intake during fasting and subsequently restarted treatment at a dose of 2.4 mg.

**Figure 1:**
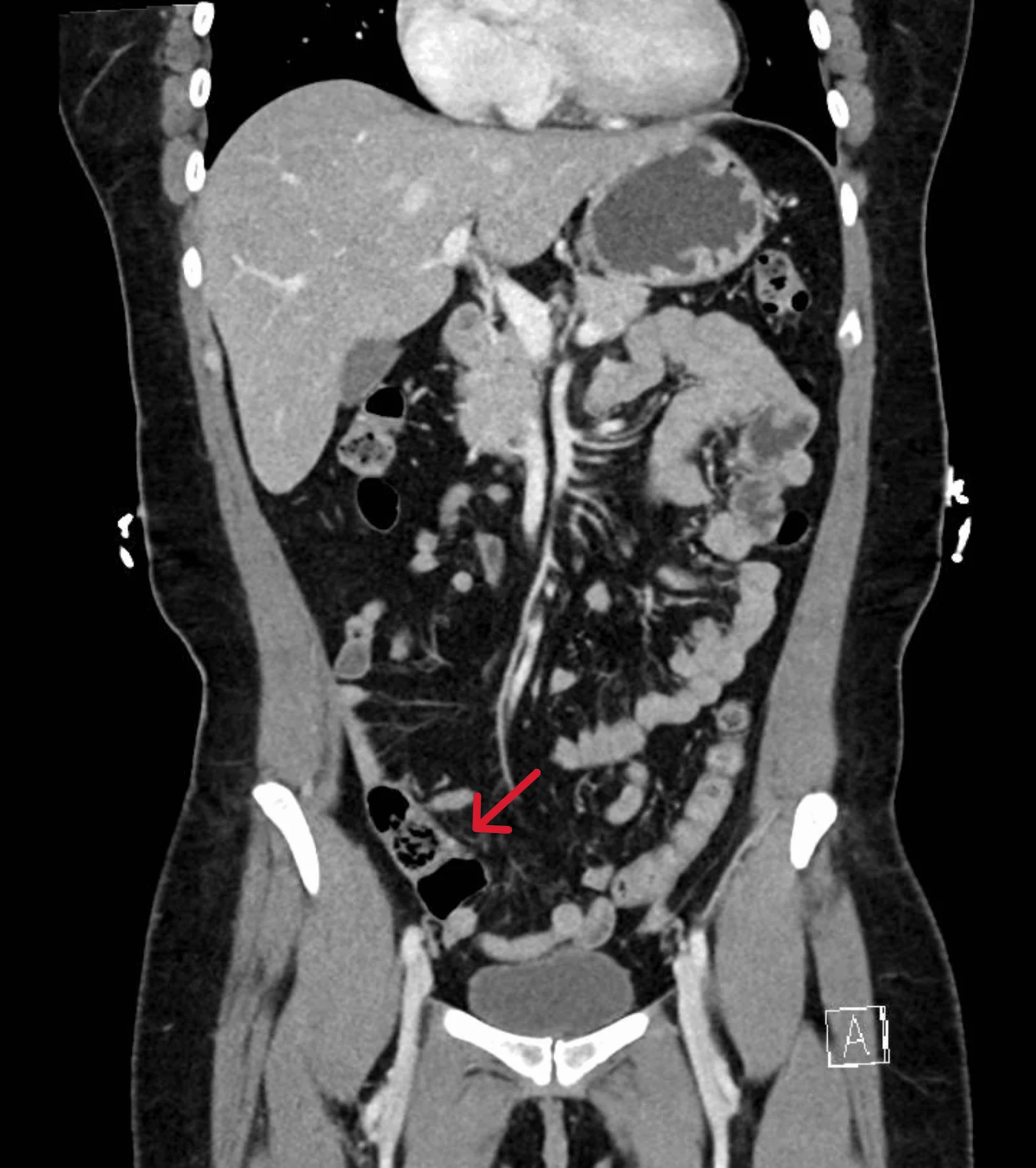
CT image of the abdomen and pelvis The CT examination demonstrated no inflammatory changes, free fluid, bowel distention, or bowel obstruction. The appendix was normal (red arrow). These findings supported the absence of an acute intra-abdominal process.

At presentation, the patient was awake and alert. Physical examination revealed warm, dry skin and moist mucous membranes. Her blood pressure was 153/107 mmHg, pulse rate was 82 beats/min, and body mass index was 29.1 kg/m². Abdominal examination revealed diffuse tenderness. Laboratory investigations showed electrolyte disturbances, including severe hypokalemia (potassium, 2.4 mmol/L; reference range, 3.5-5.1 mmol/L) and borderline low magnesium levels (magnesium, 1.8 mg/dL; reference range, 1.6-2.4 mg/dL) (Table 1). Contrast-enhanced abdominal and pelvic CT demonstrated no inflammatory changes, free fluid, bowel distention, or bowel obstruction, with a normal-appearing appendix (Figure 1).

**Table 1:** Laboratory test results HbA1c: Glycated hemoglobin

Test	Value	Reference range and units
Sodium	138	135-148 mmol/L
Potassium	2.4	3.5-5.1 mmol/L
Calcium	9.9	8.4-10.5 mg/dL
Magnesium	1.8	1.6-2.4 mg/dL
Chloride	101	98-110 mmol/L
Carbon dioxide	22	21-31 mmol/L
Blood urea nitrogen	12	7-25 mg/dL
Creatinine	0.74	0.5-1.2 mg/dL
Glucose	75	71-99 mg/dL
Glycated hemoglobin (HbA1c)	5.9	4.0-5.6%
Albumin	4.5	3.5-5.0 g/dL
Total protein	8.5	6.0-8.2 g/dL
Alanine aminotransferase	29	0-33 U/L
Alkaline phosphatase	73	30-130 U/L
Total bilirubin	0.8	<=1.2 mg/dL
Lipase	17	16-63 U/L

The patient was managed with aggressive intravenous hydration, electrolyte replacement, proton pump inhibitor therapy, antiemetics, and a clear liquid diet. Her symptoms gradually improved, and she was discharged after four days of hospitalization. Based on the temporal relationship, exclusion of alternative causes, and the patient's clinical course, a formal causality assessment using the Naranjo Adverse Drug Reaction Probability Scale [10] yielded a score of 7, indicating a probable association between semaglutide reinitiation and the observed adverse event. Reinitiation of semaglutide was planned after two weeks with cautious dose escalation (starting at 0.25 mg weekly).

## Discussion

The GLP-1 receptor agonists have become widely used in the treatment of obesity and diabetes mellitus because of their proven efficacy and additional metabolic benefits [11]. However, gastrointestinal side effects may limit treatment tolerability and, in some cases, lead to clinically significant complications [1]. In a meta-analysis of 48 randomized controlled trials involving 27,729 participants, gastrointestinal adverse events were reported in 11.66% of patients treated with GLP-1 receptor agonists, with nausea the most common (21.49%) and reduced appetite the least frequent (5.49%) [4]. Evidence from the Semaglutide Treatment Effect in People with Obesity (STEP) trials suggests that gastrointestinal adverse events related to semaglutide are usually transient and occur predominantly during the dose-escalation phase, supporting the importance of gradual dose titration to improve tolerability [12].

The use of GLP-1 receptor agonists during Ramadan is generally considered safe, although most available evidence is derived from studies in patients with type 2 diabetes [7]. Studies have demonstrated improved glycemic control and a lower risk of hypoglycemia with GLP-1 receptor agonists compared with several other glucose-lowering therapies during fasting [8]. However, gastrointestinal adverse effects, including nausea and vomiting, may increase the risk of dehydration, particularly during prolonged fasting. Therefore, initiation of GLP-1 receptor agonist therapy is recommended at least six to eight weeks before Ramadan to permit gradual dose titration and achievement of a well-tolerated maintenance dose before fasting begins [7].

Despite the generally transient nature of gastrointestinal adverse events observed in clinical trials, emerging case reports indicate that prolonged interruption of semaglutide therapy and subsequent reinitiation at higher doses may precipitate severe gastrointestinal intolerance. One previously published case described acute multisystem gastrointestinal intolerance following abrupt reinitiation of high-dose semaglutide after a two-month treatment interruption, resulting in esophagitis, metabolic derangements, and hypertensive urgency [13]. Another published case reported severe nausea and vomiting requiring medical intervention after reinitiation of target-dose semaglutide following a seven-week interruption in therapy, despite prior gradual dose escalation [14]. Our case further supports this emerging pattern, as the patient developed severe gastrointestinal intolerance requiring hospitalization after reinitiation of high-dose semaglutide following a five-week treatment interruption.

Semaglutide has an approximate subcutaneous bioavailability of 89%, with peak plasma concentrations occurring around three days after administration [6]. Steady-state levels are generally achieved after five weeks of once-weekly dosing [2]. Given its elimination half-life of approximately one week, semaglutide is expected to be largely eliminated from the circulation within five to seven weeks after the final dose [6,15]. In the present case, the five-week treatment interruption likely resulted in near-complete elimination of semaglutide, providing a pharmacokinetic rationale for reinitiating treatment with gradual dose escalation rather than immediate resumption of the maintenance dose. Recent literature has proposed practical algorithms for GLP-1 receptor agonist reinitiation after missed doses. Michelet et al. suggested that patients missing ≥2 consecutive semaglutide doses may require dose adjustment or re-titration, depending on prior gastrointestinal tolerability. Specifically, the algorithm recommends reducing the dose by one titration step after three to four missed doses, whereas reinitiation at 0.25 mg weekly is advised after interruptions of ≥5 doses [9].

Although several practical approaches for semaglutide reinitiation after treatment interruption have recently been suggested, current recommendations are still mainly derived from pharmacokinetic data and expert opinion, with limited prospective evidence available. Ramadan represents a particularly important clinical context for patient counseling because some individuals may independently discontinue GLP-1 receptor agonist therapy during fasting periods due to concerns about reduced oral intake or gastrointestinal symptoms. Clinicians should proactively counsel patients regarding continuation of therapy during Ramadan when appropriate, emphasize adequate hydration during non-fasting hours, and provide clear instructions regarding dose re-titration if treatment interruption extends beyond several weeks. Although available data generally support the use of GLP-1 receptor agonists during Ramadan, most evidence is derived from studies in patients with type 2 diabetes, and further research is needed to establish their safety and tolerability in individuals without diabetes.

## Conclusions

This case highlights the potential for severe gastrointestinal intolerance following reinitiation of high-dose semaglutide after a prolonged treatment interruption, despite previous treatment tolerability. Routine discontinuation of GLP-1 receptor agonist therapy during Ramadan is generally not recommended unless clinically indicated. If such an interruption occurs, reinitiating semaglutide at a lower dose with gradual dose escalation may help minimize gastrointestinal adverse events. Further studies are needed to establish evidence-based recommendations for semaglutide reinitiation following prolonged treatment interruptions.

## References

[1] Jalleh RJ, Plummer MP, Marathe CS (2024). Clinical consequences of delayed gastric emptying with GLP-1 receptor agonists and tirzepatide. J Clin Endocrinol Metab.

[2] Hall S, Isaacs D, Clements JN (2018). Pharmacokinetics and clinical implications of semaglutide: a new glucagon-like peptide (GLP)-1 receptor agonist. Clin Pharmacokinet.

[3] Nauck MA, Vilsbøll T, Gallwitz B, Garber A, Madsbad S (2009). Incretin-based therapies: viewpoints on the way to consensus. Diabetes Care.

[4] Xie X, Yang S, Deng S, Liu Y, Xu Z, He B (2025). Comparative gastrointestinal adverse effects of GLP-1 receptor agonists and multi-target analogs in type 2 diabetes: a Bayesian network meta-analysis. Front Pharmacol.

[5] Alhazmi A, le Roux CW (2026). Do no harm: managing nausea and vomiting in GLP-1 based obesity therapies. Front Endocrinol.

[6] Fornes A, Huff J, Pritchard RI, Godfrey M (2022). Once-weekly semaglutide for weight management: a clinical review. J Pharm Technol.

[7] Ibrahim M, Ba-Essa EM, Ahmed A (2025). Recommendations for the management of diabetes during Ramadan applying the principles of the ADA/ EASD consensus: update 2025. Diabetes Metab Res Rev.

[8] Kamrul-Hasan AB, Pappachan JM, Ashraf H, Nagendra L, Dutta D, Kuchay MS, Shaikh S (2025). Safety and efficacy of glucagon-like peptide-1 receptor agonists in individuals with type 2 diabetes mellitus fasting during Ramadan: a systematic review and meta-analysis. World J Methodol.

[9] Michelet A, Mowla M, Amo-Brown BA, Rodriguez N, Cavaretta M (2025). Missed doses, missed opportunities: readmission due to GLP-1Ra interruption inspires algorithms to improve reinitiation of therapy at discharge. Hosp Pharm.

[10] Naranjo CA, Busto U, Sellers EM (1981). A method for estimating the probability of adverse drug reactions. Clin Pharmacol Ther.

[11] Drucker DJ (2024). Efficacy and safety of GLP-1 medicines for type 2 diabetes and obesity. Diabetes Care.

[12] Wharton S, Calanna S, Davies M (2022). Gastrointestinal tolerability of once-weekly semaglutide 2.4 mg in adults with overweight or obesity, and the relationship between gastrointestinal adverse events and weight loss. Diabetes Obes Metab.

[13] Mehta J, Tai B, Frunzi J (2026). Acute systemic complications following high-dose semaglutide reinitiation in an elderly patient. Clin Case Rep.

[14] Denny O, Baron J, Albanese NP (2024). Navigating glucagon-like peptide receptor agonist reinitiation amid access barriers: an adverse drug event case report. J Pharm Pract.

[15] Overgaard RV, Delff PH, Petri KC, Anderson TW, Flint A, Ingwersen SH (2019). Population pharmacokinetics of semaglutide for type 2 diabetes. Diabetes Ther.

